# Intergenerational residential school attendance and increased substance use among First Nation adults living off-reserve: An analysis of the aboriginal peoples survey 2017

**DOI:** 10.3389/fpubh.2022.1029139

**Published:** 2023-01-20

**Authors:** Elaine Toombs, Jessie I. Lund, Aislin R. Mushquash, Christopher J. Mushquash

**Affiliations:** ^1^Department of Psychology, Lakehead University, Thunder Bay, ON, Canada; ^2^Dilico Anishinabek Family Care, Fort William First Nation, ON, Canada; ^3^Northern Ontario School of Medicine (NOSM) University, Lakehead University, Thunder Bay, ON, Canada; ^4^Thunder Bay Regional Health Sciences Centre, Thunder Bay, ON, Canada; ^5^Thunder Bay Regional Health Research Institute, Thunder Bay, ON, Canada

**Keywords:** First Nation health, Indigenous health, residential school, intergenerational trauma, substance use

## Abstract

**Introduction:**

The Truth and Reconciliation Commission of Canada (TRCC) published 94 Calls to Action in 2015 to address long-term, intergenerational effects of the residential school system, highlighting the pervasive impact of colonialism on the wellbeing of Indigenous peoples in Canada. Indeed, research with Indigenous populations in Canada has captured that prior experiences of residential schools contributes to the intergenerational transmission of mental and physical health disparities. Despite these studies, further research is needed that contextualizes the influence of residential schools within broader frameworks that consider Indigenous social determinants of health in Canada. As such, the purpose of the present study was to examine patterns of substance use and mental and physical health among individuals with a history of residential school attendance (RSA) and individuals reporting parent or two-generation (parent and grandparent) RSA.

**Method:**

Data from the Aboriginal Peoples Survey (2017), involving 10,030 First Nations individuals living off reserve, were analyzed.

**Results:**

Self-reported mental and physical health scores were significantly lower among those had attended residential schools, whose parents attended residential schools, and whose grandparents attended residential schools, when compared to those who did not. Further, family RSA was associated with increased substance use among participants, though the findings were variable based on sex and specific substance analyzed. Meanwhile, individual and family RSA was not associated with increased likelihood of a mental health diagnosis.

**Discussion:**

These findings provide additional support for how both parental and two-generation family histories of RSA are associated with individual physical and mental health outcomes. Further, these findings articulate the need for the TRCC's Calls to Action to be actually implemented, including community-based approaches that harness the strength of Indigenous people and communities who aim to close the gap in these health disparities for their children and families.

## 1. Introduction

Indigenous peoples in Canada have experienced intergenerational transmission of many detrimental physical and mental health concerns, which have been partially attributed to ongoing experiences of systemic discrimination, colonization, and cultural genocide (1–4). These intergenerational experiences of trauma have disrupted parenting practices, exacerbated untreated mental and physical health difficulties of prior generations, and contributed to disparities in Indigenous health outcomes when compared to non-Indigenous people (3, 5). Canada continues to attempt to reconcile ongoing ramifications of inequalities perpetuated by these systems, including attempts to rectify legislative actions that have reduced wellness and autonomy of Indigenous communities for generations. The Truth and Reconciliation Commission of Canada (6) published 94 Calls to Action to address long-term, intergenerational effects of the residential school system by improving child welfare, health, justice, and education systems for Indigenous peoples. For example, the 19^th^ Call to Action identifies relevant gaps and suggests methods to reduce long-term health disparities for Indigenous peoples. This includes gathering relevant data on factors that affect life expectancy within Indigenous communities, such as the presence of chronic disease, mental health, and addiction (6).

We cannot authentically understand the many health disparities experienced by Indigenous peoples without considering the ramifications of the extended history of residential schools. The residential school program in Canada, lasting from the early 1800's to 1996, removed children from their families and forced children to adopt non-Indigenous identities (6). By prohibiting the use of traditional language and cultural practices and removing children from their communities where cultural practices were often taught, many children grew up not knowing their cultural identity or how cultural practices were embedded in daily life (6). The effects of these experiences are long-lasting. Residential school attendance is associated with depressive symptoms, suicidal ideation, a history of abuse, sex work involvement, and problematic substance use (7–9).

### 1.1. Intergenerational transmission of trauma within indigenous populations

Research with Indigenous populations in Canada has described how prior experiences of residential schools has contributed to the intergenerational transmission of mental and physical health disparities (4, 10). Intergenerational trauma, first academically conceptualized by Rakoff (11) in relation to high levels of psychological distress among offspring of Holocaust survivors, describes the preliminary theories that later informed current understandings of genetic and epigenetic transmission of health outcomes between generations. This research has broadened an epigenetic understanding of how transmission of trauma can influence health across generations, including both through preconception, utero, and post-natal early child developmental effects on individual phenotypes (12). Research has since been extended globally to explain how genocides (13), famines (14), slavery (15), and refugee experiences (16) influence mental and physical health outcomes within large populations of people across generations. The relationship between residential school attendance and lower health outcomes for Indigenous people across generations remains clearly predicted. Bombay et al. (4, 10) found that family experiences of residential school attendance predict poorer health outcomes, including mental health and suicide ideation across generations. Similarly, parent residential school attendance predicts self-reported physical and health, psychological distress, suicide ideation, and suicide attempts among Indigenous individuals living off reserve (17). When childhood educational outcomes were examined, maternal residential school attendance was associated with increased school suspensions or expulsion, children being less likely to get along with teachers, and less likely to look forward to attending school (18).

Residential school attendance is associated with increased rates of mental health difficulties not only for those who have attended these institutions, but also for subsequent children and grandchildren of these survivors (4, 10). Although the last residential school closed in 1996, the effects of these practices and the continued trauma experienced within Indigenous communities are long-lasting and continue to affect the next generations of families. This type of intergenerational transmission of trauma, including how detrimental mental health symptoms are experienced across generations, has been further exacerbated experiences of colonization and cultural assimilation, decreased the transfer of culturally-useful parenting practices, and affected outstanding parent-child relationships in present day (7). Disrupted transmission of culturally-relevant parenting practices has been associated with lower emotional warmth or expressiveness by parents, increased substance abuse, and experiences of abuse or neglect by parents resulting in challenging relationships with their children and influencing the way that they parented (19). Mechanisms of action postulated in current literature to potentially explain how parental or family RSA is affiliated with mental and physical health concerns among offspring have included environmental exposure among offspring to:

- Ineffective or harsh parenting practices.
- Attachment disruptions or separation from caregiver.
- Increased exposure to caregiver mental health concerns, stress, or adverse childhood experiences (including exposure to violence, isolation, or social disadvantages).
- Community-based stressors, aggression, and racism.
- Individual isolation.
- Disruption to cultural approaches of wellness, including resilience-building or protective strategies previously used among families (4, 17, 20, 21).

To date, there is a paucity of research that has examined genetic or epigenetic differences related to families with a history of RSA. Few studies have assessed potential mechanisms of action that moderate or mediate these relationships.

The influence of residential schools on Indigenous health in Canada must be contextualized within broader frameworks that describe how these experiences intersect with Indigenous social determinants of health (22). Specific outcomes of the residential school system have created continued disparities in health status among Indigenous peoples in Canada, including *via* a loss in socio-economic status through disrupted education and employment outcomes. Educational attainment has been used to colonize, abuse, and control Indigenous individuals through forced participation in the residential school system, and educational systems continue to be sources of assimilation and discrimination for Indigenous individuals. Bolstering Indigenous engagement and participation in education systems is warranted given that higher educational attainment is associated with increased employment outcomes and higher socio-economic status which then in turn, influence health outcomes. Additional social determinants of health many Indigenous communities face include food insecurity, housing insecurity, and disrupted childhood development. For example, suicide ideation and attempts across a lifespan were disproportionally higher among individuals with lower income and food security (23). Exposure to adverse childhood experiences has been higher within Indigenous populations (24, 25) creating disruptions to typical childhood developmental processes. Such indicators, although not a proxy of intergenerational trauma, are relevant on their own accord, and therefore, may be a specific indicator of health. Given that the vast majority of residential schools in Canada were largely attended by Indigenous populations (6), residential school attendance may be a unique predictor of poor health for Indigenous populations in Canada.

Despite knowing that such experiences contribute to greater health disparities among Indigenous communities, understanding the mechanisms of actions for the transmission of such effects across generations remains limited. Epigenetic theories have focused on environmental mechanisms [including disrupted parenting/caregiving stress (26), attachment, and social learning] and biological mechanisms (including changes to typical neuroanatomical and neuroendocrine functioning and structures). For example, disruption of typical patterns of stress responses, including cortisol secretion, can create lasting influences on offspring of parents exposed to trauma (27). Multifaceted theories have incorporated such bio-psycho-social models of the influence of intergenerational stress and examined broader predictors of mental health functioning affiliated with substance use. Intergenerational transference of problematic substance use at a one to one ratio of disease transference is documented among parents and grandparents (28–30).

### 1.2. Mental health difficulties among Indigenous communities

Research on prevalence of mental health difficulties experienced within Indigenous communities is mixed. While some studies depict positive mental health outcomes [including broader life satisfaction, increased wellbeing, and absence of a mental health disorder; (31)] experienced among Indigenous individuals (67.9% of Indigenous people surveyed), when research shows disproportionally higher rates of mental health concerns within Indigenous communities. Although rates of depression, anxiety, and panic disorders can be similar among Indigenous and non-Indigenous populations (32, 33), rates of Post-Traumatic Stress Disorder (PTSD) are often reported to be higher (32–34). Indigenous youth have reported higher rates depression, anxiety, seriously considering suicide and attempting suicide (35). Suicide has been considered to be a leading cause of death for Indigenous individuals under the age of 44 [Kumar and Nahwegahbow (36) as cited in Ansloos (37)], particularly for those who identify as Inuit and among Indigenous youth.

Exposure and earlier onset of mental health concerns can be further exacerbated by barriers to accessing various health promoting social determinants, such as housing, liveable income, childcare, health care, in addition to educational and employment opportunities. For example, a population-based analysis of predictors of health found Indigenous individuals with higher education, employment, and living off-reserve were associated with higher self-reported health (38). Indigenous individuals disproportionally experience homelessness (33), incarceration (39), child welfare intervention (40, 41), all of which can amplify detrimental effects of concurrent mental health difficulties experienced across a lifespan. Indigenous populations experience a higher prevalence of Fetal Alcohol Spectrum Disorder (FASD); a recent population-based prevalence study showed statistically significant differences among Indigenous and non-Indigenous populations in Canada (42). Among Indigenous child and youth populations, the prevalence of diagnosed FASD was ~1.2%, while within non-Indigenous populations, the prevalence was 0.1% (42). In a smaller study of Indigenous children diagnosed with a FASD, the majority (80%) experienced behavioral concerns and comorbid learning disabilities (63%), and some reported involvement with the criminal justice system (12%) and alcohol use [10%; (43)]. Recent research shows that Indigenous families are also more likely to experience exposure to adverse childhood experiences (44). Such experiences can exacerbate mental health concerns experienced among communities by preventing access to timely identification, treatment, and broad-based prevention strategies.

Predictors of mental health difficulties can differ by community, and estimates that amalgamate rates of illness experienced across regions, Indigenous groups, or communities can fail to capture variation in predictors of wellness (45). Rigorous data collection protocols for Indigenous populations have been proposed to adequately track changes in suicide rates, and increase the utility of population-based data for Indigenous communities (37, 46), however to date, such approaches have yet to be implemented. As such, it remains difficult to accurately conceptualize population-based mental health trends for First Nations, or more broadly, Indigenous communities in Canada, as communities have unique strengths and challenges related to health promotion of their members. Contextualization of these experiences is required to accurately understand experiences of First Nations individuals, particularly with consideration of the underlying assumptions related to the conceptualization of mental illness, wellness, and health (37).

### 1.3. Substance use among Indigenous communities

For Indigenous populations, contextualizing high rates of problematic substance use in a way that better reflects the needs of these individuals can inform understanding of high rates of chronic physical and mental health concerns among those with substance use concerns. First Nations individuals have identified substance use as a serious concern within their communities, ranking issues from addiction and substance use as more important than both housing and employment (47). Although First Nations adults are more likely than non-Indigenous Canadians to abstain from alcohol use, those that do consume alcohol are more likely to binge drink [defined as consuming more than five drinks per occasion; FNIGC (47, 48)]. Research suggests increased substance use is also more prevalent within some First Nation populations (35, 49), particularly among Indigenous youth when rates of use were compared to non-Indigenous youth (50). A national study of youth substance use rates found that Indigenous youth were more likely to consume marijuana and alcohol, and begin use at an earlier age (50).

Substance use concerns and Substance Use Disorders (SUDs) experienced among Indigenous individuals can also be co-morbid with additional psychiatric diagnoses, including trauma and stressor, depressive, and anxiety disorders, which can complicate treatment by reducing initial treatment options and subsequent treatment outcomes (51). In one study of Indigenous adults in a residential substance use treatment, 61 percent of individuals attending treatment reported clinically significant post-traumatic stress symptoms (34). Within the same sample, 19% reported moderate or severe depressive symptoms (34), much higher than population-based samples which suggest ~7% of individuals experience clinically-significant depression symptoms (52). Recent research has begun to explore commonly co-occurring disorders affiliated with problematic substance use among Indigenous communities, including mental health disorders and chronic diseases (53, 54). A large longitudinal study found that, among Indigenous youth, rates of meeting criteria for one or more SUD was 31%, and presence of an externalizing disorder predicted increased odds of SUD diagnosis for all substances examined (alcohol and cannabis), except for nicotine (53). When use of cannabis, alcohol, e-cigarettes, and tobacco among high school students was examined, Indigenous students were significantly more likely to report polysubstance use (55). Similar trends are noted within samples of Indigenous post-secondary students (35).

These trends must be situated within culturally-relevant treatment and prevention options, including exploring predictive factors of higher use. The health burden of substance use across a lifespan can also be disproportionally impactful among Indigenous populations, given that Indigenous peoples have unique experiences that can contribute to broader health difficulties. For example, Indigenous peoples were five times more likely to die from opioid use in 2017 (56), which has likely been exacerbated during the COVID-19 pandemic (57). Among First Nations individuals who have reported opioid misuse, 91% reported attending a residential school, 73% had experienced a crisis or natural disaster, 67% reporting that a friend or family member had attempted suicide, and 61% reported trauma from the completed suicide deaths of family and friends (58). First Nations adults seeking treatment at an Indigenous-led health center have reported disproportionately higher rates of adverse childhood experiences, including early exposure to abuse, neglect, and household dysfunction (44). As such, pathways of promoting Indigenous health and wellbeing across a lifespan are both multi-faceted and complex, particularly when intersecting social determinants of health and exposure to life stressors are considered.

## 2. The current study

### 2.1. Study purpose

Prevention strategies employed with Indigenous populations may address intergenerational aspects of substance use transmission by identifying shared predictors among these groups and integrating such findings with previous knowledge of harm resulting from the residential school system. Experiences of historical trauma have contributed to the intergenerational transmission of health outcomes that cannot be resolved without consideration of protective factors that foster resilience among Indigenous communities, culturally-relevant interventions, and understanding mechanisms of transmission across populations (59, 60). Given that previous intergenerational pathways to mental health among large samples of Indigenous individuals in Canada have been documented (4, 10), it is possible that such predictors across generations can be extended to improve an overall understanding of higher substance use among Indigenous communities.

The purpose of this study was to identify patterns of substance use among individuals with a history of residential school attendance as well as individuals reporting parent or two-generation (parent and grandparent) residential school history. Although previous literature has explored intergenerational patterns of mental health difficulties, no studies to date have explored whether attendance at a residential school is associated with increased substance use difficulties among offspring using a large, population-based sample. The current study aimed to assess if residential school attendance was associated with risky substance use, among First Nations individuals living off-reserve.

### 2.2. Study hypotheses

We hypothesized those with a history of individual, parent and grandparent residential school attendance would self-report lower physical and mental health ratings when compared to those who had not attended a residential school (Hypothesis 1). We also hypothesized that increased frequency of substance use would be associated with history of residential school attendance among First Nation individuals living off-reserve. Specifically, when each substance was entered as a dependent variable, we hypothesized that individual, parent-only, and two-generation (parent and grandparent) residential school attendance would be associated with increased odds of daily tobaccos use, daily cannabis use, alcohol use, and illicit substance use.

Secondary analyses also explored related hypotheses predicting that individual, parent-only, and two-generation residential school attendance would be associated with increased odds of being diagnosed with an anxiety disorder, a mood disorder, or any mental health disorder, in addition to history of suicide contemplation, or attempts (in the past year or across a lifetime).

## 3. Method

### 3.1. Participants

For the purpose of this study, data from First Nations individuals living off reserve were analyzed (Table 1). In accordance with release of data in accordance with Statistics Canada policies, each individual case in the Aboriginal Peoples Survey (APS, 2017) was adjusted to represent a broader population using a seven-step weighting method. An adjusted weight was applied to each case and accounted for non-response, partial response, and a post-stratification that corresponded each unit with population estimates based strata of Aboriginal status, region, and age group (61). The final sample initially consisted of *n*= 10,030 individuals who self-identified as First Nation and was weighted to represent 491,010 individuals in these analyses.

**Table 1:** Participant demographics.

**Demographic**		**Frequency**	**%**
Sex	Male	221,200	45.05
	Female	269,810	54.95
Age		M = 40.4 (sd = 17.07)	
Life stage	Youth (age 15–19)	62,570	12.74
	Adult	389,810	87.26
Marital status	Single	207,470	42.25
	Married	140,990	140,990
	Living common law	79,940	16.28
	Separated	16,220	3.3
	Divorced	31,210	6.36
	Widowed	15,130	3.08
Place of residence	Rural (under 1 000)	112,930	23.00
	Small population center (1,000–29,999)	107,750	21.95
	Medium population center (30,000–99,999)	72,860	14.84
	Large urban population center (100,000 or more)	197,410	40.21
Household type	Two-generation household	254,680	51.87
	Three or more-generation household	29,130	5.93
	Skip-generation household	10,810	2.2
	Other household type	195,790	39.88
Highest attained level of education	Grade 8 or lower	24,050	4.90
	Some secondary education	64,130	13.06
	Secondary school diploma	73,560	14.98
	Some post-secondary	85,540	17.42
	Post-secondary diploma	150,460	30.64
	Bachelor's degree	34,170	6.96
	Degree above bachelor level	14,730	3.00
Employment status	Employed	267,820	54.54
	Unemployed	43460	8.85
	Not in labor force	177,710	36.19
Estimated 2016 total personal income	< $, 000	71,210	14.5
	$5,000–9,999	34,470	7.02
	$10,000–19,999	52,630	10.72
	$20,000–29,999	45,850	9.34
	$30,000–39,999	33,330	6.79
	$40,000–49,999	27,550	5.61
	$50,000–69,999	48,430	9.86
	$70,000 and over	35,200	7.17
Residential school attendance	Individual attendance	31,570	8.52
	Parent attendance	120,640	26.78
	Grandparent attendance	163,100	44.86

### 3.2. Measures

#### 3.2.1. Data set

Data from the APS 2017 was accessed with permission from the Canadian Research Data Centre Network (CRDCN) and analyzed at the Research Data Center at McMaster University using Stata 17. The 2017 APS is a national survey of Indigenous peoples in Canada, specifically First Nations individuals living off-reserve, Métis, and Inuit populations. Across the five iterations of the APS, occurring approximately every 5 years, the survey has focused on social and economic conditions of Indigenous peoples in Canada. The Lakehead University Research Ethics Board (REB) provided an exemption to REB approval, as this study used de-identified secondary data. The data analytic plan was approved by representatives from the CRDCN, and was drafted to comply with Canadian Institutes of Health Research, Natural Sciences and Engineering Research Council of Canada, Social Sciences and Humanities Research Council of Canada (62) and Ownership Control Assess Possession [OCAP^TM^; (63)] principals when possible.

All data were aggregated, and no individual scores for any respondent were reported. Congruent with Statistics Canada data regulations for the APS (2017), if a sample size within a particular sub-population of variables was below 10, results were not reported. Place of residence was calculated by using participant-reported description of population size, and was not described for any individual community, to preserve confidentiality and to adhere to OCAP™ (63) principles related to individual community sovereignty regarding research goals, approaches, and dissemination of results.

#### 3.2.2. Variables

For the purposes of this study, variables depicting demographical characteristics (age, sex, socio-economic status, education level, and household members, among others), and self-report ratings of mental health, physical health, and employment status were used from relevant APS variables. Data was selected for those participants who identified as First Nation to the question, “Are you First Nations, Métis, or Inuk?”. Binary dummy variables were derived from specific substance use questions in the APS 2017. Increased frequency of substance use was defined as daily tobacco or marijuana use, consuming 5 or more alcoholic drinks in one period (once a week or more), or off-label prescription drug use or street drug use (once a month or more).

##### 3.2.2.1. Tobacco

Daily tobacco was entered as 1, while both other frequencies (“occasionally” or “not at all”) were ranked as 0.

##### 3.2.2.2. Marijuana

Similarly, daily or almost marijuana use was coded 1, while the remaining four categories (“at least once a week,” “at least once a month,” “less than once a month,” or “not at all”) were coded as 0.

##### 3.2.2.3. Alcohol

With respect to alcohol use, self reported use of either “once a week” or “more than once a week” was coded as 1, while other frequencies (“2–3 times a month,” “once a month,” “less than once a month,” or “never”) were coded as 0.

##### 3.2.2.4. Illicit substance use

Due to lower responses for either prescription and street drug use frequency, these categories were collapsed together and responses of “at least once a month,” “at least once a week, and “daily or almost daily” were coded as 1, while use of “less than a month” or “not at all” were coded as 0.

### 3.3. Analysis procedure

Independent *t*-tests were used to describe differences in mean self-reported mental and physical health among groups of individuals who had attended residential schools and those who had not (Hypothesis 1). We also aimed to completed exploratory analyses related to sex differences among physical and mental health ratings, however did not have a hypothesis predicting any anticipated differences.

Logistic regressions (Hypothesis 2) compared frequency of use for each substance among those with an individual history of RSA, those with single generation (parent) history of RSA, and those reporting both grandparent and parent (two-generation) RSA history. Within each of these regressions, both age and sex were entered as covariates, and for analyses with single or two-generation RSA, individual's own history of RSA was also entered as a covariate. Among these analyses, daily tobacco and cannabis use was associated with individual, parent, and two-generation RSA.

In an attempt to reduce the likelihood of a multiplicity error from non-adjusted analyses within the current study, the analyses of mental health disorders and suicide ideation were considered to be a secondary goal of this study. These comparisons should be considered as exploratory.

## 4. Results

Self-reported mental and physical health scores (Table 2) were significantly lower among those had attended residential schools, whose parents attended residential schools, and whose grandparents attended residential schools, when compared to those who did not. This was predicted in Hypothesis 1. When these self-reported health ratings were compared by sex in an exploratory analysis, male participants reported both better mental and physical health outcomes than female participants.

**Table 2:** Independent *t*-tests of self-reported mental and physical health of individual, parent, and grandparent RSA.

			***m***	**Bootstrapped *SE***	**CI (lower)**	**CI (upper)**	***t***	***p***
Self-reported mental health	Sex	Male	2.67	0.026	2.62	2.72	−7.67	0.000
		Female	2.41	0.024	2.40	2.45		
	Individual RSA	RSA	2.39	0.069	2.26	2.53	−2.77	0.006
		Non-RSA	2.58	0.021	2.54	2.62		
	Parent RSA	RSA	2.42	0.035	2.35	2.48	−4.06	0.000
		Non-RSA	2.58	0.020	2.54	2.62		
	Grandparent RSA	RSA	2.40	0.032	2.34	2.47	−5.84	0.000
		Non-RSA	2.64	0.023	2.60	2.67		
Self-reported physical health	Sex	Male	2.42	0.027	2.37	2.48	9.92	0.000
		Female	2.26	0.023	2.20	2.30		
	Individual RSA	RSA	2.17	0.037	2.09	2.24	−4.98	0.000
		Non-RSA	2.42	0.020	2.38	2.50		
	Parent RSA	RSA	2.30	0.035	2.23	2.37	−5.95	0.000
		Non-RSA	2.41	0.024	2.37	2.47		
	Grandparent RSA	RSA	2.30	0.035	2.23	2.37	−3.01	0.003
		Non-RSA	2.42	0024	2.37	2.47		

### 4.1. Hypothesis 2: Substance use frequency

Results of hypothesis 2 are found in Table 3.

**Table 3:** Predictors of substance use frequency including individual, parent, and two-generation RSA.

		**Individual RSA**				**Single generation RSA (Parent)**				**Two generation RSA** **(Parent** + **Grandparent)**			
		**z** ^*^	**OR**	**CI** **(-)**	**CI (**+**)**	**z** ^*^	**OR**	**CI** **(-)**	**CI (**+**)**	**z** ^*^	**OR**	**CI** **(-)**	**CI (**+**)**
Daily tobacco use	Age	−4.35^a^	0.98	0.984	0.994	−4.2^a^	0.99	0.982	0.994	−2.91^a^	0.99	0.985	0.997
	Sex (female)	0.31	1.03	0.870	1.211	0.15	1.01	0.849	1.209	−0.44	0.96	0.783	1.167
	Constant	−3.41	0.63	0.478	0.819	−3.58	0.58	0.427	0.780	−4.00	0.50	0.351	0.699
	Individ. RSA	3.04^b^	1.58	1.175	2.112	1.81	1.38	0.973	1.960	2.08^c^	1.51	1.025	2.244
	Parent RSA					3.34^1^	1.43	1.159	1.765				
	Two gen RSA									2.91^a^	1.22	1.066	1.238
Alcohol consumption of 5 or more drinks, once a week or more	Age	0.08	1.00	0.993	1.008	−0.60	1.00	0.990	1.006	−1.58	0.99	0.983	1.001
	Sex (Female)	−3.49^a^	0.61	0.466	0.807	−3.00^a^	0.64	0.481	0.858	−2.58^b^	0.66	0.476	0.903
	Constant	−9.21	0.11	0.107	0.234	−8.05	0.17	0.109	0.260	−5.98	0.21	0.127	0.352
	Individ. RSA	0.05	0.63	0.626	1.636	−1.03	0.77	0.479	1.257	−0.62	0.84	0.490	1.447
	Parent RSA					1.34	1.22	0.911	1.642				
	Two gen RSA									0.46	1.05	0.862	1.272
Daily cannabis use	Age	−9.50^a^	0.95	0.941	0.961	−9.40^a^	0.95	0.937	0.958	−7.84^a^	0.95	0.938	0.962
	Sex (Female)	−4.57^a^	0.52	0.389	0.686	−4.19^a^	0.52	0.383	0.706	−3.79^a^	0.51	0.365	0.725
	Constant	0.14	1.03	0.644	1.659	0.12	1.03	0.611	1.746	−0.41	0.88	0.471	1.638
	Individ. RSA	2.62^a^	1.95	1.184	3.222	1.73	1.69	0.932	3.054	1.64	1.75	0.896	3.416
	Parent RSA					2.41^c^	1.52	1.081	2.149				
	Two gen RSA									2.38^c^	1.33	1.052	1.689
Monthly or greater prescription or illicit drug abuse	Age	−5.08^a^	0.96	0.938	0.972	−4.76^a^	0.95	0.932	0.971	−3.06^a^	0.96	0.943	0.987
	Sex (Female)	−1.95	0.61	0.374	1.003	−2.19^c^	0.56	0.336	0.941	−2.28^c^	0.51	0.290	0.912
	Constant	−3.09	0.25	0.101	0.598	−2.91	0.20	0.065	0.587	−3.51	0.11	0.032	0.378
	Individ. RSA	−0.19	0.93	0.437	1.979	−1.31	0.58	0.251	1.319	−0.88	0.68	0.285	1.615
	Parent RSA					4.22^a^	3.02	1.810	5.071				
	Two gen RSA									4.64^a^	1.85	1.426	2.392

a *p* ≥ 0.001.

b *p* ≥ 0.01.

c *p* > 0.05.

* Significance tests of constant values not reported.

#### 4.1.1. Tobacco use

Respondents with an individual history of RSA were 1.6 times more likely to use tobacco daily and 2.6 times to use cannabis daily compared to those without a history. Among those with parent and two-generation RSA history, respondents were 1.4x more likely to use cannabis daily if they had a parent who attended a residential school, and 2.9x more likely to use cannabis daily if both a parent and a grandparent attended.

#### 4.1.2. Cannabis use

Similarly, with respect to cannabis use frequency, individuals with a personal history of RSA were 2.6 times more likely to use cannabis daily, however were 2.4 times more likely if a parent had attended a residential school, and 2.4x more likely if a parent and a grandparent had attended. In initial analyses of individual RSA, males were 1.9 times more likely to use cannabis daily compared to female participants, which was significant at a 0.001 level.

#### 4.1.3. Alcohol use

When alcohol use frequency was analyzed using logistic regressions, frequency of use was not predicted by individual, parent, or two-generation residential school attendance, however similar to cannabis use frequency, males were more likely to consume 5 or more drinks a week compare to females. In initial analyses examining individual RSA, males were 1.6 times more likely to have higher alcohol consumption.

#### 4.1.4. Illicit substance use

Illicit substance use frequency was not significantly predicted by an individual history of RSA, however was associated with both parent and two-generation RSA. Among those who reported a parent history of RSA, individuals were 3.0 times more likely to engage in monthly or greater illicit substance or prescription drug abuse. For individuals with two-generation RSA, they were 1.9 times more likely to fall in the higher illicit use category.

### 4.2. Mental health disorder and suicide-specific secondary analyses

Contrary to our hypotheses, logistic regression results (Table 4) did not show any statistically significant relationship among these diagnostic categories and any type of RSA when age and sex were entered as covariates. Within initial analyses, female participants were 2.5 times more likely to be diagnosed with an anxiety disorder and 2.1 times more likely to have a diagnosis of a mood disorder, however there were no sex differences found for the broader category of having any type of mental health disorder diagnosis.

**Table 4:** Predictors of mental health disorder diagnoses including individual, parent, and two-generation RSA.

		**Individual RSA**				**Single Generation RSA (Parent)**				**Two Generation RSA** **(Parent** + **Grandparent)**			
		**z** ^*^	**OR**	**CI** **(-)**	**CI (**+**)**	**z** ^*^	**OR**	**CI** **(-)**	**CI (**+**)**	**z** ^*^	**OR**	**CI** **(-)**	**CI (**+**)**
Diagnosis of any mental health disorder	Age	−3.82^a^	0.97	0.953	0.985	−3.42^a^	0.97	0.959	0.989	−3.49^a^	0.97	0.950	0.986
	Sex (female)	−1.79	0.60	0.342	1.051	−1.91	0.57	0.319	1.014	−0.96	0.72	0.369	1.404
	Constant	−1.80	0.47	0.204	1.068	−2.59	0.36	0.167	0.781	−1.28	0.528	0.198	1.409
	Individ. RSA	1.15	1.72	0.683	4.350	1.20	1.87	0.673	5.209	1.53	2.50	0.778	8.035
	Parent RSA					−0.12	0.96	0.506	1.821				
	Two gen RSA									−1.29	0.72	0.441	1.185
Diagnosis of an anxiety disorder	Age	−5.99^a^	0.97	0.973	0.986	−6.07^a^	0.98	0.971	0.985	−6.43^a^	0.97	0.967	0.982
	Sex (female)	8.31^a^	2.54	2.041	3.170	8.03^a^	2.57	2.040	3.233	6.91^a^	2.48	1.919	3.213
	Constant	−5.80	0.34	0.239	−0.492	−5.36	0.35	0.241	0.517	−3.83	0.42	0.271	0.656
	Individ. RSA	0.43	1.09	0.740	1.600	0.22	1.05	0.697	1.571	0.13	1.03	0.644	1.657
	Parent RSA					1.15	1.15	0.908	1.448				
	Two gen RSA									1.24	1.10	0.944	1.292
Diagnosis of a mood disorder	Age	−3.62^a^	0.99	0.983	0.995	−3.67^a^	0.99	0.982	0.995	−2.98^b^	0.99	0.982	0.996
	Sex (female)	7.33^a^	2.11	1.729	2.579	7.40^a^	2.23	1.804	2.763	5.63^a^	2.07	1.606	2.667
	Constant	−8.51	0.24	0.176	0.338	−8.19	0.22	0.162	0.327	−7.08	0.21	0.139	0.327
	Individ. RSA	−0.73	0.88	0.624	1.240	−1.34	0.77	0.531	1.126	−1.25	0.75	0.483	1.174
	Parent RSA					1.03	1.12	0.899	1.404				
	Two gen RSA									1.54	1.12	0.968	1.304

a *p* ≥ 0.001.

^b^*p* ≥ 0.01.

^c^*p* > 0.05.

* Significance tests of constant values not reported.

A lifetime history and frequency in the past year of both suicide contemplation and attempts was also analyzed with respect to individual, parent, and two-generation RSA. These hypotheses were tested using logistic regression, however results (Table 5) were inconsistent across these analyses. Only two statistically significant relationships emerged; parent RSA was associated with lifetime suicide contemplation (OR = 2.2) and two-generation RSA was associated with having a suicide attempt in the last year (OR = 2.8).

**Table 5:** Predictors of suicide contemplation and attempts including individual, parent, and two-generation RSA.

		**Individual RSA**				**Single generation RSA (Parent)**				**Two generation RSA** **(Parent** + **Grandparent)**			
		**z** ^*^	**OR**	**CI** **(-)**	**CI (**+**)**	**z** ^*^	**OR**	**CI** **(-)**	**CI (**+**)**	**z** ^*^	**OR**	**CI** **(-)**	**CI (**+**)**
Contemplated suicide in lifetime	Age	−4.49^a^	0.98	0.978	0.991	−4.18^a^	0.98	0.977	0.992	−4.30^a^	0.98	0.973	0.990
	Sex (female)	4.72^a^	1.62	1.328	1.986	4.78^a^	1.67	1.352	2.060	3.90^a^	1.64	1.28	2.09
	Constant	−6.22	−0.34	0.238	0.474	−5.95	0.30	0.204	0.448	−4.27	0.35	0.223	0.573
	Individ. RSA	1.11	1.23	0.852	1.783	0.54	1.12	0.734	1.719	0.65	1.18	0.722	1.914
	Parent RSA					2.21^c^	1.31	1.032	1.669				
	Two gen RSA									1.25	1.11	0.943	1.305
Contemplated suicide in past year	Age	−1.29	0.99	0.975	1.005	−1.16	0.99	0.975	1.007	−0.56	0.99	0.977	1.013
	Sex (female)	−0.01	1.00	0.665	1.500	0.28	1.06	0.691	1.638	0.54	1.15	0.702	1.869
	Constant	−1.83	0.48	0.219	1.052	−1.89	0.44	0.192	1.031	−2.35	0.32	0.121	0.826
	Individ. RSA	0.04	1.01	0.519	1.984	0.10	1.04	0.490	2.199	−0.02	0.99	0.428	2.288
	Parent RSA					−0.19	0.96	0.605	1.513				
	Two gen RSA									0.41	1.06	0.790	1.434
Suicide attempt in lifetime	Age	1.44	1.01	0.997	1.021	−0.19	1.00	0.972	1.024	0.61	1.00	0.990	1.020
	Sex (Female)	1.78	1.44	0.963	2.161	1.43	1.37	0.890	2.094	0.59	1.15	0.725	1.825
	Constant	−2.28	0.47	0.245	0.899	−2.01	0.495	0.249	0.982	−1.62	0.51	0.221	1.153
	Individ. RSA	1.74	1.92	0.922	4.006	1.31	1.73	0.762	3.944	1.45	1.98	0.785	5.015
	Parent RSA					1.40	1.35	0.888	2.037				
	Two gen RSA									1.54	1.25	0.941	1.663
Suicide attempt in last year	Age	−0.44	0.99	0.970	1.019	−0.19	1.00	0.972	1.024	1.29	1.02	0.991	1.044
	Sex (female)	−1.37	0.53	0.214	1.317	−0.65	0.71	0.249	2.001	−0.52	0.73	0.228	2.348
	Constant	1.13	0.14	0.032	0.644	−2.61	0.08	0.012	0.532	−4.23^a^	0.01	0.002	0.101
	Individ. RSA	1.13	1.84	0.638	5.316	0.52	1.35	0.435	4.208	0.08	1.05	0.318	3.486
	Parent RSA					1.22	1.84	0.693	4.868				
	Two gen RSA									2.76^a^	2.24	1.262	3.963

a *p* ≥ 0.001.

^b^*p* ≥ 0.01.

c *p* > 0.05.

* Significance tests of constant values not reported.

## 5. Discussion

This study explored how individual, parent, and two-generation (having both a parent and a grandparent) histories of RSA influence frequency of tobacco, cannabis, alcohol, and other illegal substances. We also used exploratory analyses to examine similar trends for prevalence of any type of mental health disorder, and more specifically, anxiety and mood disorders. Study analyses demonstrated that, overall, family RSA was associated with increased substance use among individuals, however results were somewhat inconsistent across substances and dependent on who in the family had attended a residential school (individual, parent, or both parent and grandparent). When age and sex were entered as covariates, having both a parent and a grandparent attend a residential school was associated with increased odds of individual daily tobacco use, daily cannabis use, or misusing prescriptions or street drugs monthly or more. Having both a parent and a grandparent attend a residential school was not associated with increased odds of alcohol use (consuming more than five standard drinks in one setting, once a week or more), which was a similar finding when both parent-only and individual RSA were examined. Individual RSA was associated with increased odds of daily tobacco and daily cannabis use, while parent-only RSA was associated with daily tobacco use, daily cannabis use, and monthly or greater street drug or prescription drug misuse.

The current study has supported previous literature that associated intergenerational RSA with individual health outcomes among offspring (1, 4, 10), with a focus on substance use frequency and mental health diagnoses. We examined one pathway, within a series of relatively complex relationships, that described how various types of family histories of RSA can influence present-day substance use among individuals. Results suggest that such experiences are associated with increased substance use across most substances examined in the current study. Further examination of these nuances may inform broader conceptualizations of the intergenerational transmission of mental health difficulties using a bio-psycho-social model (64) as a relevant framework. For example, when biological differences were examined in hair samples and compared with exposure to life stressors, Indigenous women had significantly higher cortisone levels when compared to non-Indigenous women. These relationships trended in the opposite direction for Indigenous men, such that Indigenous men had significantly lower cortisone levels (65). It is likely that various predictors of the intergenerational transmission of trauma, substance use, and additional mental health difficulties vary within intersecting biological, social, and psychological determinants (66).

Current findings were generally consistent with previous trends describing sex differences among mental health, substance use, and suicide ideation within Indigenous populations (67). Although family or individual RSA was not associated with increased odds of frequent alcohol use when sex differences were examined, male participants were more likely to engage in higher frequency alcohol use and daily cannabis use. Female participants in our sample were more likely to have a diagnosis of an anxiety or mood disorder. With respect to a lifetime history of suicide contemplation, female participants were also more likely to have contemplated suicide in their lifetime, although male participants were more likely to have attempted suicide in the last year. These results suggest that there continue to be gendered experiences of mental health difficulties within Indigenous samples in Canada, which can trend similar to non-Indigenous population data.

Previous literature has explored how higher rates of childhood maltreatment and ACEs are associated with increased rates of specific psychiatric disorder (44) in a First Nation sample; However, in the current study, individual and family RSA was not associated with increased likelihood of a mental health diagnosis. Similar to previous data analyzed from the 2012 iteration of the APS (68), individual residential school attendance was not associated with increased likelihood of an anxiety disorder in our analyses. It is likely that barriers to receiving health care, including perceived stigma of and access to specialized care providers, including psychological assessment, may potentially bias these findings. Increased substance use may also mask mood or anxiety symptoms experienced by an individual, particularly when motives of use are considered. Given motives of use (social, coping, enhancement, and conformity) can differ among First Nations individuals (69), those who consume for coping reasons may not necessarily report higher anxiety and depression. Lastly, given that colonization has impacted the wellbeing and mental health of all Indigenous peoples (not solely those who have a history/family history of RSA), it may be difficult to clearly delineate RSA effects with the use of binary mental health diagnosis variables, whereas symptom severity measures may provide more information. Further research regarding pathways to increased prevalence rates of particular psychiatric disorders within Indigenous communities may be a better approach to addressing these questions, particularly when such experiences are examined across a lifespan.

### 5.1. Public health, clinical, and policy implications

Indigenous peoples in Canada have consistently reported the wide spread impact of residential schools on individual, family, and community wellbeing (6). The detrimental effects of RSA across generations of Indigenous families are well-documented, and the 94 Calls to Action aim to rectify the disproportionate social, health, and cultural influence of government mandated colonization, discrimination, and marginalization (6). Federal government leaders have even gone so far to relay that all TRCC Calls to Action will be implemented within current federal leadership terms, however limited steps to achieving this aspirational goal have been completed to date (45). Future efforts can explore potential mechanisms of authentically implementing the TRCC Calls to Action, including necessary public health interventions, policy adaption, and reconciliation efforts that can improve Indigenous health outcomes related to histories of RSA previously described in the final report. It is likely that culturally-relevant public health interventions that address housing instability, poverty, disproportionate criminal justice involvement, and exposure to ACEs can increase wellbeing among Indigenous families, particularly among individuals experiencing substance use and mental health difficulties (70).

Culturally-relevant clinical interventions that simultaneously address both co-morbid substance use and trauma are required, particularly those that embed mechanisms to address family-related trauma. The effect of intergenerational mental health difficulties on offspring may be exacerbated when one is consistently exposed to those with similar concerns. It can be challenging to treat individual mental health difficulties when their most proximal environment is in conducive to support individual change within treatment, no matter how motivated an individual may be. Given that First Nation individuals living on-reserve are more likely to live in crowded housing, and those households are most frequently multi-generational (71), there may be a clustering effect of increased mental health difficulties when older generations in the household have a history of residential school attendance. Family-based therapy or simultaneous implementation of multi-generational treatment within a household may facilitate meaningful change, however given the intensive nature of such treatment, access to such programs remains limited. Although embedding family supports and family-based approaches are more common in child or youth-focused programs, such models could be extended to adults as well to address broader family systems.

Future health policies can provide more resources to develop and assess efficacy for intergenerational treatment of mental health concerns within Indigenous families. Currently mental health funding and services available to Indigenous populations are primarily individually-focused. For example, non-insured health benefits for mental health counseling, available to only registered “Status Indians” in Canada, is billed for each individual client and does not provide any type of family coverage (72). A focus on family-based benefits, that could comprehensively include an entire family structure, regardless of treaty status, may be one down-stream solution to address ongoing mental health concerns within family units. Such interventions could likely prevent ongoing exacerbation of concerns or reduce effects of caregiving stress on offspring in such households.

### 5.2. Limitations and future directions

General limitations of the APS include generalizability of the survey respondents to broader the population and response bias given the method of survey administration. The APS is only completed with First Nations individuals living off-reserve and therefore, cannot be generalized to all First Nations individuals in Canada, particularly those living on-reserve (61). Given that the APS is completed predominately by telephone, in-person, or a combination of these methods from individuals who had completed the long-form of 2016 Canadian census, the sample does not include individuals who are incarcerated, living with no fixed address, or who could not be contacted using computer-assisted questionnaires completed in-person or by telephone. Another limitation is that there may be a response bias within some questions of the APS. In accordance with RDC guidelines, any cross-section of the data resulting in < 10 individual cases cannot be analyzed or reported. Self-reported rates of mental health diagnoses or substance use frequency may also be under-reported in health research. This is a relevant gap in the current sample and will affect the representativeness of the study results as there are disproportionately more Indigenous people in Canada incarcerated and homeless (73).

It was not possible in this study to explore specific experiences of RSA, including experiences of child maltreatment, duration of time spent within residential schools, and various protective factors such as individual level factors (such as access to culture, prosocial relationships, community engagement) or broader social determinants of health (including housing, health care, income stability). We did not explore predictors of poly-substance use (often a more clinically-relevant measure) which could be examined in future studies. We also could not contextualize who in the individual's family was their primary caregiver, which is particularly relevant given that many Indigenous individuals live in skip-generation households. One limiting assumption made within the current study, and within many studies which examine intergenerational RSA, is that parental RSA is somehow more influential on an individual's health outcomes than grandparent RSA, and that two-generational RSA exposure may contribute to a more cumulative effect on individual mental health. Undoubtedly, being directly raised by an individual with a history of RSA, may negatively influence individual outcomes, regardless of if they are one's biological parent or grandparent. Future research can either control for or directly examine the mediating role of primary caregiver, rather than formally defined biological relationships of grandparent or parent, on individual mental health outcomes.

This study could not assess how moderators and mediators of parent or grandparent substance use potentially influence the relationships among experiencing household dysfunction as a child and individual substance use. For example, although Indigenous individuals are more likely to engage in risky alcohol use, they are also more likely to be entirely abstinent from alcohol when compared to national Canadian samples (47). The current analyses did not contextualize aspects that promote resilience to reduce the negative health effects of RSA on substance use frequency, mental health, or suicide ideation. By examining mediators and moderators of increased health and wellbeing among those with histories of family RSA, it is possible that such studies can inform broader public health approaches to addressing the continued harm from RSA within Indigenous communities. For example, although current retribution efforts have aimed to provide financial compensation for residential school survivors in one approach to reconcile these histories, such efforts do not necessarily address subsequent mental or physical health difficulties from abuse, neglect, isolation, death, and disrupted relationships with family, community, and culture. It is possible that for First Nation individuals seeking treatment for experiences of trauma and subsequent mental health disorders, particularly with a family history of similar concerns, such complexity can impede initial engagement in services, treatment outcomes, and availability of treatment options.

Future studies can explore aspects of resilience and wellbeing among individuals with prior family histories of RSA who do not identify as experiencing substance use difficulties. There are likely pathways that have mitigated the detrimental effects of RSA, including individual, family, community, and cultural strengths. Although individual community has unique strengths, concerns, and health needs, it is possible that findings from previous community-based interventions could potentially be adapted to other communities or regions with careful community review and consultation. Prospective studies that explore longitudinal health outcomes across a lifespan are warranted, including those that assess specific social determinants of Indigenous health, including histories of child welfare involvement, lower educational attainment, and food insecurity (74). Interventions that address the intergenerational disruptions to cultural connectedness and ceremony, language use, parenting practices, and overall health or wellbeing can also be explored through such studies.

### 5.3. Conclusion

Understanding the mechanisms underlying the relationship between family histories of RSA and Indigenous mental health outcomes is undoubtedly complex, and cannot necessarily be contextualized using cross-sectional survey designs. Previous research has explored both theoretical and quantifiable relationships related to the transmission of mental health difficulties, and most studies reflect a need to expand current understanding of the mechanisms of action within both specific mental health disorders and broader concerns (27, 75–78). Studies highlight the need to disentangle genetic differences from non-genetic ones (including cultural transmission), focus on paternal and maternal experiences, and the influence of maladaptive parenting or care-giver stress (79). Given that offspring of have partially shared genetic make-up from their biological parents, and often, a shared environment, it can be difficult to confidently determine predictors of transmission or factors influencing heritability.

The current study could not associate how specific experiences within residential schools could contribute to further mental health difficulties across generations, however did describe broader trends across families. A history of RSA can be conceptualized as a series of exposure to cumulative traumas, however the exposure of specific experiences of adversity vary at an individual level. Although the history of these institutions is unfortunately shared across all Indigenous communities in Canada, individuals differ in their response to exposure. Despite the last residential school closing in 1996, the unfortunate legacy of these institutions will be long-lasting in regards to the detrimental influence on the mental health and wellbeing of generations of Indigenous families. Community-based approaches are being developed to address harm from residential schools, which harness the resilience and tenacity of Indigenous people and communities who aim to close the gap in these health disparities for their children and families.

## Data availability statement

Publicly available datasets were analyzed in this study. This data can be found at: https://crdcn.ca/data/aboriginal-peoples-survey/.

## Ethics statement

Ethical review and approval was not required for the study on human participants in accordance with the local legislation and institutional requirements. Written informed consent to participate in this study was provided by the participants' legal guardian/next of kin.

## Author contributions

ET completed all analyses and reporting of results. ET and JL drafted this manuscript. AM and CM reviewed final drafts. All authors contributed to the conceptualization of this manuscript.
